# Supplementary material for: Origins of the central Macaronesian psyllid lineages (Hemiptera; Psylloidea) with characterization of a new island radiation on endemic Convolvulus floridus (Convolvulaceae) in the Canary Islands
Source: PLoS One. 2024 Jan 26;19(1):e0297062. doi: 10.1371/journal.pone.0297062 (PMC10817144; doi:10.1371/journal.pone.0297062)

Supporting Information – Figure S2

Origins of the central Macaronesian psyllid lineages (Hemiptera; Psylloidea) with characterization of a new island radiation on endemic *Convolvulus floridus* (Convolvulaceae) in the Canary Islands

Saskia Bastin[1], J. Alfredo Reyes-Betancort[2], Felipe Siverio de la Rosa[1] and Diana M. Percy[3*]

[1]Instituto Canario de Investigaciones Agrarias, Unidad de Protección Vegetal, C/ El Boquerón s/n, 38200, La Laguna, Tenerife, Spain.

E-mail: bastin.saskia@hotmail.be; https://orcid.org/0000-0001-9307-7223

E-mail: fsiverio@icia.es; https://orcid.org/0000-0002-8886-414X

[2]Instituto Canario de Investigaciones Agrarias, Jardín de Aclimatación de La Oratava, C/ Retama 2, 38400 Puerto de la Cruz, Tenerife, Spain.

E-mail: areyes@icia.es; https://orcid.org/0000-0003-0732-3219

[3]Botany Department and Biodiversity Research Centre, University of British Columbia, Vancouver, British Columbia, Canada.

E-mail: diana.percy@ubc.ca; https://orcid.org/0000-0002-0468-2892

*Corresponding author E-mail: diana.percy@ubc.ca

Figure S2. Bayesian analysis using cox1 data for *Percyella*, *Drepanoza*, and select outgroup taxa from Group A (Percy et al. 2018) [52]. Full length sequences were used for the *Percyella* samples (sequence length shown with sample code).

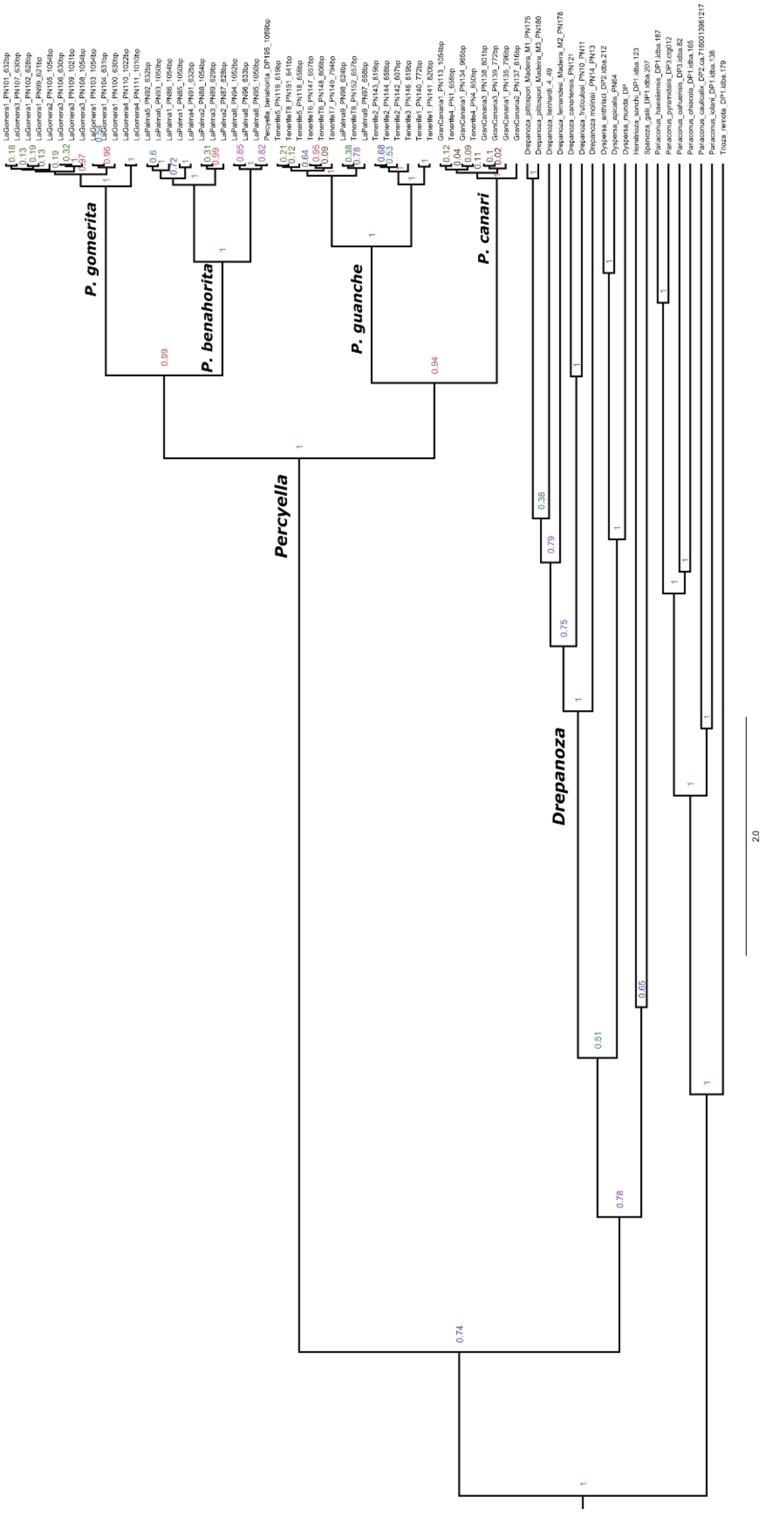

Supplement: S2 Fig — Full length sequences were used for the Percyella samples (sequence length shown with sample code). (PDF) [file pone.0297062.s002.pdf]
