# Supplementary material for: Origins of the central Macaronesian psyllid lineages (Hemiptera; Psylloidea) with characterization of a new island radiation on endemic Convolvulus floridus (Convolvulaceae) in the Canary Islands
Source: PLoS One. 2024 Jan 26;19(1):e0297062. doi: 10.1371/journal.pone.0297062 (PMC10817144; doi:10.1371/journal.pone.0297062)
Supplement: S1 Table — Molecular data: cox1: cytochrome oxidase 1, cytb: cytochrome B. (PDF) [file pone.0297062.s004.pdf]

Supporting Information – Table S1

Origins of the central Macaronesian psyllid lineages (Hemiptera; Psylloidea) with characterization of a new island radiation on endemic *Convolvulus floridus* (Convolvulaceae) in the Canary Islands

Saskia Bastin[1], J. Alfredo Reyes-Betancort[2], Felipe Siverio de la Rosa[1] and Diana M. Percy[3*]

[1]Instituto Canario de Investigaciones Agrarias, Unidad de Protección Vegetal, C/ El Boquerón s/n, 38200, La Laguna, Tenerife, Spain.

E-mail: bastin.saskia@hotmail.be; https://orcid.org/0000-0001-9307-7223

E-mail: fsiverio@icia.es; https://orcid.org/0000-0002-8886-414X

[2]Instituto Canario de Investigaciones Agrarias, Jardín de Aclimatación de La Oratava, C/ Retama 2, 38400 Puerto de la Cruz, Tenerife, Spain.

E-mail: areyes@icia.es; https://orcid.org/0000-0003-0732-3219

[3]Botany Department and Biodiversity Research Centre, University of British Columbia, Vancouver, British Columbia, Canada.

E-mail: diana.percy@ubc.ca; https://orcid.org/0000-0002-0468-2892

*Corresponding author E-mail: diana.percy@ubc.ca

Supporting Information Table S1. Summary of all non-Macaronesian psyllid taxa for which molecular data was generated.
Molecular data: cox1: cytochrome oxidase 1, cytb: cytochrome B.

Species	Collection locality	Collection date	Host plant	Molecular data
Agonoscena cisti (Puton, 1882)	Spain	19 April 2016	*Pistacia* sp.	cox1, cytb
Agonoscena targionii (Lichtenstein, 1874)	Spain	19 April 2016	*Pistacia* sp.	cox1, cytb
Arytaina genistae (Latreille, 1804)	Canada	2014	*Cytisus scoparius*	cox1, cytb
Cacopsylla alaterni (Foerster, 1848)	France	15-20 April 1990	No information	cox1, cytb
Cacopsylla alaterni (Foerster, 1848)	Spain	23 Dec 1997, 24 March 1998, 19 April 2016	*Rhamnus alaternus*	cox1, cytb
Cacopsylla myrthi (Puton, 1876)	Israel	07 May 2010	"conifers"	cox1, cytb
Cacopsylla myrthi (Puton, 1876)	Spain	23 Dec 1997	*Rhamnus alaternus*	cox1, cytb
Cacopsylla rhamnicola (Scott, 1876)	UK	2003	*Rhamnus catharticus*	cox1, cytb
Drepanoza lienhardi (Burckhardt, 1981)	Israel	06 May 2010	*Lycium schweinfurthii*	cox1
Euphyllura olivina (Costa, 1839)	Spain	May 2021	*Olea* europapaea	cox1
Strophingia ericae (Curtis, 1835)	UK	1994	No information	cytb
