# Supplementary material for: Origins of the central Macaronesian psyllid lineages (Hemiptera; Psylloidea) with characterization of a new island radiation on endemic Convolvulus floridus (Convolvulaceae) in the Canary Islands
Source: PLoS One. 2024 Jan 26;19(1):e0297062. doi: 10.1371/journal.pone.0297062 (PMC10817144; doi:10.1371/journal.pone.0297062)
Supplement: S2 Table — Abbreviations: H: El Hierro, P: La Palma, G: La Gomera, T: Tenerife, C: Gran Canaria, F: Fuerteventura, L: Lanzarote. (PDF) [file pone.0297062.s005.pdf]

Supporting Information – Table S2

Origins of the central Macaronesian psyllid lineages (Hemiptera; Psylloidea) with characterization of a new island radiation on endemic *Convolvulus floridus* (Convolvulaceae) in the Canary Islands

Saskia Bastin[1], J. Alfredo Reyes-Betancort[2], Felipe Siverio de la Rosa[1] and Diana M. Percy[3*]

[1]Instituto Canario de Investigaciones Agrarias, Unidad de Protección Vegetal, C/ El Boquerón s/n, 38200, La Laguna, Tenerife, Spain.

E-mail: bastin.saskia@hotmail.be; https://orcid.org/0000-0001-9307-7223

E-mail: fsiverio@icia.es; https://orcid.org/0000-0002-8886-414X

[2]Instituto Canario de Investigaciones Agrarias, Jardín de Aclimatación de La Oratava, C/ Retama 2, 38400 Puerto de la Cruz, Tenerife, Spain.

E-mail: areyes@icia.es; https://orcid.org/0000-0003-0732-3219

[3]Botany Department and Biodiversity Research Centre, University of British Columbia, Vancouver, British Columbia, Canada.

E-mail: diana.percy@ubc.ca; https://orcid.org/0000-0002-0468-2892

*Corresponding author E-mail: diana.percy@ubc.ca

Supporting Information Table S2. Summary of Macaronesian endemic *Convolvulus* species (Gobierno de Canarias 2023) [13] surveyed during this study with distribution of *Convolvulus*-feeding psyllids. Abbreviations: H: El Hierro, P: La Palma, G: La Gomera, T: Tenerife, C: Gran Canaria, F: Fuerteventura, L: Lanzarote.

***Convolvulus* species**	**Distribution**	**Island surveyed**	**Psyllid species**
Convolvulus canariensis	H,P,G,T,C	P,G,T	*Drepanoza canariensis*
Convolvulus caput-medusae	C,F	C	none
Convolvulus floridus	P,G,T,C,F,L	P,G,T,C,L	all four *Percyella* spp. (none in Lanzarote)
Convolvulus fruticulosus subsp. *fruticulosus*	H,P,T	T	*Drepanoza fruticulosi*
Convolvulus fruticulosus subsp. *glandulosus*	C	–	[?] not surveyed
Convolvulus lopezsocasii	L	L	none
Convolvulus perraudieri	T,C	T	none
Convolvulus scoparius	H,P,G,T,C	T	none
Convolvulus subauriculatus	G	G	none
Convolvulus volubilis	G,T	G	none
