# Supplementary material for: Origins of the central Macaronesian psyllid lineages (Hemiptera; Psylloidea) with characterization of a new island radiation on endemic Convolvulus floridus (Convolvulaceae) in the Canary Islands
Source: PLoS One. 2024 Jan 26;19(1):e0297062. doi: 10.1371/journal.pone.0297062 (PMC10817144; doi:10.1371/journal.pone.0297062)
Supplement: S3 Table — (PDF) [file pone.0297062.s006.pdf]

Supporting Information – Table S3

Origins of the central Macaronesian psyllid lineages (Hemiptera; Psylloidea) with characterization of a new island radiation on endemic *Convolvulus floridus* (Convolvulaceae) in the Canary Islands

Saskia Bastin[1], J. Alfredo Reyes-Betancort[2], Felipe Siverio de la Rosa[1] and Diana M. Percy[3*]

[1]Instituto Canario de Investigaciones Agrarias, Unidad de Protección Vegetal, C/ El Boquerón s/n, 38200, La Laguna, Tenerife, Spain.
E-mail: bastin.saskia@hotmail.be; https://orcid.org/0000-0001-9307-7223
E-mail: fsiverio@icia.es; https://orcid.org/0000-0002-8886-414X
[2]Instituto Canario de Investigaciones Agrarias, Jardín de Aclimatación de La Oratava, C/ Retama 2, 38400 Puerto de la Cruz, Tenerife, Spain.
E-mail: areyes@icia.es; https://orcid.org/0000-0003-0732-3219
[3]Botany Department and Biodiversity Research Centre, University of British Columbia, Vancouver, British Columbia, Canada.
E-mail: diana.percy@ubc.ca; https://orcid.org/0000-0002-0468-2892

*Corresponding author E-mail: diana.percy@ubc.ca

Supporting Information Table S3. Host plant genera of the Central Macaronesian native psyllids with the number of Canarian and Macaronesian endemic and non-endemic species (Gobierno de Canarias, 2023) [13].

Host plant genus	Canarian endemic species	Macaronesian endemic species	Non-endemic species
Pistacia			2
Ruta	4		2
Cistus	8		2
Picconia	1		
Olea	1		1
Erica	1	1	
Chamaecytisus	1		?
Spartocytisus	2		
Teline	9		
Adenocarpus	3		
Salix		1	
Rhamnus	2	1	1
Gymnosporia	2		
Retama	1		
Convolvulus	9		5
Laurus	1		1
Withania	1		2
