# Supplementary material for: Origins of the central Macaronesian psyllid lineages (Hemiptera; Psylloidea) with characterization of a new island radiation on endemic Convolvulus floridus (Convolvulaceae) in the Canary Islands
Source: PLoS One. 2024 Jan 26;19(1):e0297062. doi: 10.1371/journal.pone.0297062 (PMC10817144; doi:10.1371/journal.pone.0297062)
Supplement: S4 Table — (PDF) [file pone.0297062.s007.pdf]

Supporting Information – Table S4

Origins of the central Macaronesian psyllid lineages (Hemiptera; Psylloidea) with characterization of a new island radiation on endemic *Convolvulus floridus* (Convolvulaceae) in the Canary Islands

Saskia Bastin[1], J. Alfredo Reyes-Betancort[2], Felipe Siverio de la Rosa[1] and Diana M. Percy[3*]

[1]Instituto Canario de Investigaciones Agrarias, Unidad de Protección Vegetal, C/ El Boquerón s/n, 38200, La Laguna, Tenerife, Spain.

E-mail: bastin.saskia@hotmail.be; https://orcid.org/0000-0001-9307-7223

E-mail: fsiverio@icia.es; https://orcid.org/0000-0002-8886-414X

[2]Instituto Canario de Investigaciones Agrarias, Jardín de Aclimatación de La Oratava, C/ Retama 2, 38400 Puerto de la Cruz, Tenerife, Spain.

E-mail: areyes@icia.es; https://orcid.org/0000-0003-0732-3219

[3]Botany Department and Biodiversity Research Centre, University of British Columbia, Vancouver, British Columbia, Canada.

E-mail: diana.percy@ubc.ca; https://orcid.org/0000-0002-0468-2892

*Corresponding author E-mail: diana.percy@ubc.ca

Supporting Information Table S4. Summary of the endemic central Macaronesian psyllid species and lineages (if in situ diversification occurred), indicating number of species per lineage, host plant, and the distribution and host plant of the closest sister group (where relationship is resolved with 80% or greater bootstrap support).

Species/lineage	Species number	Host plant family	Host plant species/lineage	Sister group	Sister group distribution	Sister group host plant family	Host plant species/genus for sister group
Agonoscena atlantica	1	Anacardiaceae	*Pistacia atlantica*	*Agonoscena pistaciae*	Mediterranean	Anacardiaceae	*Pistacia* spp. including *Pistacia atlantica*
Agonoscena sinuata	1	Rutaceae	*Ruta pinnata*	*Agonoscena targionii*	Mediterranean	Anacardiaceae	*Pistacia* ssp.
Lisronia echidna	1	Cistaceae	*Cistus monspeliensis*	*Rhinocola aceris*	Europe/Mediterranean	Sapindaceae	*Acer* spp.
Euphyllura canariensis	1	Oleaceae	*Picconia excelsa*	unresolved	–	–	–
Euphyllura confusa	1	Oleaceae	*Olea europaea*	*Euphyllura olivina*	Mediterranean	Oleaceae	*Olea europaea*
Megadicrania tecticepss	1	Oleaceae	*Olea* spp.	unresolved	–	–	–
Strophingia lineage	4	Ericaceae	*Erica* spp.	*Strophingia ericae*	Europe/Mediterranean	Ericaceae	*Erica cinerea*
Arytaina lineage I	3	Fabaceae	*Chamaecytisus proliferus* ssp.	*Arytaina* spp.	Europe/Mediterranean	Fabaceae	*Adenocarpus* spp., *Chamaecytisus* spp.
Arytaina lineage II	2	Fabaceae	*Spartocytisus* spp.	*Arytaina* spp.	Europe/Mediterranean	Fabaceae	*Adenocarpus* spp., *Chamaecytisus* spp.
Arytainilla serpentina	1	Fabaceae	*Spartocytisus filipes*	*Arytainilla spartiophila*	Europe/Mediterranean	Fabaceae	*Cytisus scoparius*
Arytinnis lineage	18	Fabaceae	*Teline* spp., *Adenocarpus* spp., *Chamaecytisus* spp., *Genista* sp.	*Livilla* ssp.	Europe/Mediterranean	Fabaceae	*Retama, Genista, Laburnum, Chamaecytisu, Cytisus, Lembotropis*
Cacopsylla atlantica	1	Salicaceae	*Salix canariensis*	*Cacopsylla saliceti*	Europe/Mediterranean	Salicaceae	*Salix* spp.
Cacopsylla crenulatae	1	Rhamnaceae	*Rhamnus crenulata*	*Cacopsylla myrthi*	Mediterranean	Rhamnaceae	*Rhamnus alaternus*

Cacopsylla lineage I	2	Rhamnaceae	*Rhamnus glandulosa*	*Cacopsylla rhamnicola*	Europe/Mediterranean	Rhamnaceae	*Rhamnus* spp.
Diaphorina gonzalezi	1	Celastraceae	*Gymnosporia cassinoides*	*Diaphorina gymnosporiae*	South Asia	Celastraceae	*Gymnosporia royleana, G. spinosa*
Livilla monospermae	1	Fabaceae	*Retama rhodorhizoides*	*Livilla* spp. including *Livilla retamae*	Europe/Mediterranean	Fabaceae	*Retama, Genista, Laburnum, Chamaecytisu, Cytisus, Lembotropis*
Drepanoza lineage I	3	Convolvulaceae	*Convolvulus* lineage I *Whitania aristata*	unresolved	–	–	–
Drepanoza lineage II	2	Convolvulaceae	*Pittosporum coriaceum*	unresolved	–	–	–
Lauritrioza laurisilvae	1	Lauraceae	*Laurus novocanariensis*	*Lauritrioza alacris*	Mediterranean	Lauraceae	*Laurus nobilis*
Percyella lineage	4	Convolvulaceae	*Convolvulus* lineage II (*C. floridus*)	*Spanioza galii*	Europe/Mediterranean	Rubiaceae	*Ambrosia, Asperula, Cruciata, Galium, Rubia, Scherardia, Sherardia*
