# Supplementary material for: Origins of the central Macaronesian psyllid lineages (Hemiptera; Psylloidea) with characterization of a new island radiation on endemic Convolvulus floridus (Convolvulaceae) in the Canary Islands
Source: PLoS One. 2024 Jan 26;19(1):e0297062. doi: 10.1371/journal.pone.0297062 (PMC10817144; doi:10.1371/journal.pone.0297062)
Supplement: Attachment — Submitted filename: Comments to the authors.pdf [file pone.0297062.s009.pdf]

I have read the impressive work of Bastin and colleagues titled "Origins of the central Macaronesian psyllid lineages (Hemiptera; Psylloidea) with characterization of a new island radiation on endemic *Convolvulus floridus* (Convolvulaceae) in the Canary Islands", and I absolutely loved it.

The work not only tackles the always-timely topic of island biogeography using the superfamily Psylloidea as a model, but also uses a variety of molecular methods to better understand psyllid phylogeny, colonisation events, and radiations.

The specimens used for this work comprise most of the Macaronesian species, providing a near-complete set of information, only slightly hindered by the low resolution highlighted at some points (either due to lack of species or to the use of specific genetic markers, as the authors clearly state).

All in all, I think this work is extremely important not only for the general understanding of a scarcely studied insect group such as psyllids, but also as a model to study other insect groups and their island radiation.

I have only a few very minor comments and suggestions before recommending this work for publication:

- Line 195: either add the authority to all species names, or remove it from *Drepanoza montanetana.*
- Line 196: since this is a relatively specific list, I would substitute "some" with the actual number of species.
- Line 232: remove ")".
- Line 238: Please, specify ethanol %.
- Line 240-242: Just for clarity and consistency, I would be more specific when discussing the markers used. The expression "mitochondrial region" is a bit vague and quite non-specific. Either use "a fragment of the subunit I of the mitochondrial gene *cytochrome oxidase*" (with the gene name italicised), or perhaps include a reference to the "barcoding region", for which I would probably add a citation to Hebert et al. 2003 (as the first paper on the use of DNA barcoding).
- Line 246: Same as above for cytb.
- Line 257: Wrong symbol used for "°C".
- Line 257: For consistency, either name all the different PCR steps (i.e., denaturation, annealing and extension) or remove "annealing temperature".
- Line 304: MRCS = Most Recent Common Ancestor.
- Line 333: Perhaps use "performed" instead of "done".
- Line 359: Remove "added".
- Line 398: "Included" (instead of "include") for consistency.
- Line 420: Add hyphen in "Lauraceae-feeding species".
- Line 438: I would add something on the lines of "included in this study" after "The only member of *Livilla*", since you proceed to explain that this species groups with other *Livilla* spp. This should read: "The only member of *Livilla* included in this study..".
- Line 440-443: This sentence is a bit convoluted, probably due to the number of commas and insets. If you could rephrase or break it down into two sentence, that would probably facilitate reading.
- Line 457: "any particular taxon" instead of "a particular taxon"
- Line 607: remove comma after "phylogeny". It should read "in the original backbone phylogeny (e.g., for *Megadicrania*) can be problematic".
- Line 631: add comma after "incumbent advantage)".

- Line 642: change “*and”* (italicised) to “and” (normal).
- Line 729: I understand your point, and I think this is an extremely interesting topic. I understand so far host switching across diverse hosts has been considered a very rare phenomenon, but I also think in some cases this could still be hidden under the fact we don’t have much genetic data available. For example, the work of Ouvrard’s and colleagues (2015) was still almost entirely devoid of any phylogenetic base, which is getting clearer and clearer only since the work of Percy and colleagues (2018). Additionally, it happens often that researchers tend to work on smaller datasets, sometimes starting from the host plant association and collecting psyllids associated with a given plant genus, only. This can strongly bias our understanding of the major host switches, until we have a clearer picture also thanks to DNA analysis.
  For example, some important host switchings (across a wide number of host plant families) have been reported from the New Zealand psyllid species of the genus *Powellia,* and recently confirmed to have originated from a single colonisation + radiation event. The present study is probably only the third or fourth work examining in depth the relationships between psyllids and their hosts in the context of island colonisation and radiation events. See: Dale 1985 and Martoni et al. 2023. (Please, note this is just an FYI since I find this topic extremely interesting, and these don’t require to be referenced in this work!).
  Martoni F, Bulman SR, Piper AM, Pitman A, Taylor GS, Armstrong KF (2023) Insect phylogeny structures the bacterial communities in the microbiome of psyllids (Hemiptera: Psylloidea) in Aotearoa New Zealand. PLoS ONE 18(5): e0285587.
  Dale PJ (1985) A review of the Psylloidea (Insecta: Hemiptera) of the New Zealand subregion. Ph.D. Thesis, University of Auckland. 1985. Available from: http://hdl.handle.net/2292/1694.
- Line 730: not sure if this is a requirement from the journal, but these references are not in order of publication.
