# Supplementary material for: Origins of the central Macaronesian psyllid lineages (Hemiptera; Psylloidea) with characterization of a new island radiation on endemic Convolvulus floridus (Convolvulaceae) in the Canary Islands
Source: PLoS One. 2024 Jan 26;19(1):e0297062. doi: 10.1371/journal.pone.0297062 (PMC10817144; doi:10.1371/journal.pone.0297062)
Supplement: Attachment — Submitted filename: Response to Reviewers_PERCY_9Dec2023.docx [file pone.0297062.s010.docx]

**Response to Reviewers**	9th December 2023

Dear Editor,

Please consider our revised submission titled:

**Origins of the central Macaronesian psyllid lineages (Hemiptera; Psylloidea) with characterization of a new island radiation on endemic *Convolvulus floridus* (Convolvulaceae) in the Canary Islands**

for publication in PLOS One.

Below we provide detailed responses to the Reviewers’ comments.

Thank you for your consideration.

Diana Percy

Dept. of Botany and Biodiversity Research Centre, University of British Columbia, Canada

diana.percy@ubc.ca

=======

**Response to Reviewers**

**Reviewer 1:**

The reviewer made 25 comments in the edited manuscript PDF and these are addressed below with the Reviewer's comment (if given) and Line numbers referring to those in the Reviewer's edited PDF document:

1) Line 43. alphabetical

**RESPONSE**: We have changed the list of plant names to be alphabetical as suggested. We have also done the same for another instance at lines 733-734 in original submission.

2) Line 52. I find "atypical" a bit confusing. There are many examples of psyllid diversification on the same host species, e.g. Colophorina, Jataiba and Mitrapsylla on Copaifera langsdorffii, Platycorypha on Hymenaea courbaril or Queiroziella on Mimosa scabrella. Rephrase.

**RESPONSE**: As the reviewer points out, there are examples of species in the same genus apparently diversifying on the same host plant, and we thank the reviewer for providing some good examples which we now reference. But the fact remains that a pattern indicating a process of speciation on the same host species is not the case for the majority of psyllid diversification events that are known, i.e., where it has been shown that sister taxa are found on the same plant species and not simply that there are a number of species in the same genus found on the same host plant. We therefore opt to retain our use of "atypical" in this instance, but we have added a sentence using some of the examples suggested by the reviewer to the Introduction (fourth paragraph) to clarify this point. The sentence added is given below in Response to comment 7).

3), 4), 5) Line 61.

**RESPONSE**: We have deleted the 3 words as suggested.

6) Line 116. Perhaps it is worth mentioning that there are several examples of closely related psyllid species associated sympatrically with the same host species, e.g. Mitrapsylla spp. on Copaifera langsdorffii, Calophya spp. on Schinus longifolia or S. polygama, Cacopsylla spp. on Crataegus laevigata or Queiroziella spp. on Mimosa scabrella.

**RESPONSE**: We have now included mention of these other studies/taxa as suggested and we have added context - see in the following Response for comment 7).

7) Line 120-121. See comment in abstract

**RESPONSE**: Again, the point here is that the majority of the speciation events determined to be sister taxa involve a host switch, so we therefore retain the argument here that a radiation on the same host is atypical in psyllids. As per the earlier comment/response on this (see 2) and 6) above) we have added a sentence mentioning other groups that may represent sympatric speciation on the same host, however in most cases this has not explicitly been tested. We therefore retain our wording here. The added sentence to the Introduction (fourth paragraph) to clarify this point is:

“…and although a number of psyllid genera in continental regions are known to have multiple species occurring on the same host plant species (e.g., *Arytainilla*, *Bactericera*, *Cacopsylla*, *Calophya*, *Mitrapsylla*, *Queiroziella*), most of these examples have not explicitly tested whether the psyllid taxa sharing the same host are sister species and whether the speciation events may have occurred sympatrically or allopatrically,”."

8) Line 184. Shouldn't this be a dash?

**RESPONSE**: We have replaced the hyphen with a dash.

9) Line 199. Better change to "adventive" or "exotic" throughout the manuscript.

**RESPONSE**: The terms "introduced" and "adventive" are sometimes used synonymously, and the difference appears to lie in whether introduced taxa have become fully established or not. As the extent of establishment differs across the non-native species treated in our paper, we had used the term "introduced" broadly to differentiate from "native". However, although we do not think there is any real misunderstanding in using "introduced", we have changed our use to "adventive". This change has been made across the manuscript and in Fig 1.

10) Line 203. dash

**RESPONSE**: We have changed to dash as suggested.

11) Line 204. dash

**RESPONSE**: We have changed to dash as suggested.

12) Line 215. Lauterer was

**RESPONSE**: We have added a space.

13) Line 231. species

**RESPONSE**: We have changed specific to species as suggested.

14) Line 232-233. This is not very useful. Is there a literature reference? Are there differences to the two sources mentioned above?

**RESPONSE**: Yes, there are differences between the publically available databases in regards to accepted plant names of some of the host plant taxa, and we have given the references for the databases consulted in the previous sentence. As we are only pointing out here that these differences exist for some of the host plant names, we do not think it is appropriate to elaborate further on the status of the plant taxonomy. However, we now clarify that the differences exist for *Chamaecystisus* at both species and subspecies levels.

15) Line 270.

**RESPONSE**: We have deleted "superfamily" as suggested.

16) Line 315-317. Use a dash for ranges throughout the manuscript.

**RESPONSE**: We have replaced hyphens with dashes for age ranges.

17) Line 360. Better "exotic" or "adventive". Change everywhere.

**RESPONSE**: We have changed "introduced" to "adventive" - see also our comment on this under 9) above.

18) Line 377. This sounds as Arytaina is exclusively in the Canary Islands. Rephrase.

**RESPONSE**: We have rephrased this sentence and the following one, as suggested.

19) Line 420-421. I think this is not true. Older sources (cited in Psyl'list) are probably incorrect.

**RESPONSE**: We have removed this part of the sentence, as suggested.

20) Line 480-481. The closest relatives of Lisronia echidna are L. splendida and varicicosta, both continental species assciated with Cistus spp. You would predict, that any Lisronia species is associated with Cistus, but you could not predict the host of Lisronia withing the context of the phylogeny of Rhinocolinae genera. Reword.

**RESPONSE**: We thank the reviewer for pointing out the host associations for other continental Lisronia taxa and we have modified the sentence (and added a reference), as suggested, to reflect this as follows:

"...although not included in our sampling, there are two continental *Lisronia* species in the Mediterranean region known from *Cistus* host plants, and in particular *L. varicicosta* (Hodkinson & Hollis) is considered to be the most likely sister taxon of the endemic Canarian *L. echidna* (Burckhardt and Lauterer, 1989) and both species occur on the same widespread host plant (*C. monspeliensis*) that is found in Macaronesia, western Mediterranean, and North Africa. Therefore, although strong support grouping *Lisronia* and *Rhinocola* implies a phylogenetically wide host switch occurred between these host plant families at some point, the colonization of the Canary Islands by *Lisronia* most likely follows the common pattern of colonization of a familiar host plant."

Reference added:

Burckhardt, D. & Lauterer, P. (1989) Systematics and biology of the Rhinocolinae (Homoptera: Psylloidea). Journal of Natural History, 23:3, 643-712. doi: 10.1080/00222938900770371

21) Line 519. Perhaps it is worth mentioning here that D. lienhardi develops on Lycium (Solanaceae).

**RESPONSE**: We thank the reviewer for pointing out that the host genus for *Drepanoza lienhardi* is not given, and we have amended this sentence, as suggested.

22) Line 566. cannot

**RESPONSE:** Spelling correction made, as suggested.

23) Line 627. ch

**RESPONSE**: Spelling correction made, as suggested.

24) Line 648. cannot

**RESPONSE**: Spelling correction made as suggested.

25) Line 655. Not true as the continental relatives are also associated with Cistus spp.

**RESPONSE**: We have modified this sentence to correct the interpretation.

===========

**Reviewer 2:**

The reviewer made 21 comments and these are listed and addressed below with the Line numbers given referring to those in the original submission:

- Line 195: either add the authority to all species names, or remove it from Drepanoza montanetana.

**RESPONSE**: We have left this unchanged as the authority here is given only for the species not already mentioned in the ms (with authority already given on first mention for the other species).

- Line 196: since this is a relatively specific list, I would substitute “some” with the actual number of species.

**RESPONSE**: We have added the exact number (15).

- Line 232: remove “)”.

**RESPONSE**: We have not removed this parenthesis, as it encloses correctly - the sentence has double brackets due to a reference within the bracketed text.

- Line 238: Please, specify ethanol %.

**RESPONSE**: We have specified the % (70-85%).

- Line 240-242: Just for clarity and consistency, I would be more specific when discussing the markers used. The expression “mitochondrial region” is a bit vague and quite non-specific. Either use “a fragment of the subunit I of the mitochondrial gene cytochrome oxidase” (with the gene name italicised), or perhaps include a reference to the “barcoding region”, for which I would probably add a citation to Hebert et al. 2003 (as the first paper on the use of DNA barcoding).

**RESPONSE**: We now use the word "fragment" rather than "region" and add "subunit" to the gene name. For a citation, we have included here a recent review of barcoding primers for psyllids (which also cites the Hebert et al. 2003 reference mentioned by the reviewer).

- Line 246: Same as above for cytb.

**RESPONSE**: Likewise, we use "fragment" here.

- Line 257: Wrong symbol used for “°C”.

**RESPONSE**: We have corrected the symbol.

- Line 257: For consistency, either name all the different PCR steps (i.e., denaturation, annealing and extension) or remove “annealing temperature”.

**RESPONSE**: We have already provided names for the PCR steps, therefore no changes have been made.

- Line 304: MRCS = Most Recent Common Ancestor.

**RESPONSE**: We have corrected the word order.

- Line 333: Perhaps use “performed” instead of “done”.

**RESPONSE**: The word change has been made, as suggested.

- Line 359: Remove “added”.

**RESPONSE**: This word has been removed.

- Line 398: “Included” (instead of “include”) for consistency.

**RESPONSE**: The word change has been made, as suggested.

- Line 420: Add hyphen in “Lauraceae-feeding species”.

**RESPONSE**: We have added a hyphen here and to another instance on Line 432 (legume-feeding).

- Line 438: I would add something on the lines of “included in this study” after “The only member of Livilla”, since you proceed to explain that this species groups with other Livilla spp. This should read: “The only member of Livilla included in this study..”.

**RESPONSE**: We have clarified this sentence to indicate this is the only "island" member of Livilla and that it groups "with continental" Livilla. Hence, it is not the only Livilla included in our study.

- Line 440-443: This sentence is a bit convoluted, probably due to the number of commas and insets. If you could rephrase or break it down into two sentence, that would probably facilitate reading.

**RESPONSE**: We have broken this into two sentences.

- Line 457: “any particular taxon” instead of “a particular taxon”

**RESPONSE**: We prefer to leave the wording as is in this instance, as it conveys our meaning best.

- Line 607: remove comma after “phylogeny”. It should read “in the original backbone phylogeny (e.g., for Megadicrania) can be problematic”.

**RESPONSE**: We have removed the comma as suggested.

- Line 631: add comma after “incumbent advantage)”.

**RESPONSE**: We have added a comma as suggested.

- Line 642: change “and” (italicised) to “and” (normal).

**RESPONSE**: We have made the change as suggested.

- Line 729: I understand your point, and I think this is an extremely interesting topic. I understand so far host switching across diverse hosts has been considered a very rare phenomenon, but I also think in some cases this could still be hidden under the fact we don’t have much genetic data available. For example, the work of Ouvrard’s and colleagues (2015) was still almost entirely devoid of any phylogenetic base, which is getting clearer and clearer only since the work of Percy and colleagues (2018). Additionally, it happens often that researchers tend to work on smaller datasets, sometimes starting from the host plant association and collecting psyllids associated with a given plant genus, only. This can strongly bias our understanding of the major host switches, until we have a clearer picture also thanks to DNA analysis.

For example, some important host switchings (across a wide number of host plant families) have been reported from the New Zealand psyllid species of the genus Powellia, and recently confirmed to have originated from a single colonisation + radiation event. The present study is probably only the third or fourth work examining in depth the relationships between psyllids and their hosts in the context of island colonisation and radiation events.

See: Dale 1985 and Martoni et al. 2023. (Please, note this is just an FYI since I find this topic extremely interesting, and these don’t require to be referenced in this work!).

**RESPONSE**: The reviewer makes several interesting points regarding sampling and taxon focus in interpretating processes involving near and wide host switching, and to incorporate these, we have added a couple of sentences at the end of the paragraph to broaden the scope of our discussion accordingly, and included an added citation:

“However, it is worth noting that, although less commonly encountered and apparently more prevalent in particular psyllid taxa, there are instances where phylogenetically wide host switching (between plant families) is the dominant process during diversification (e.g., *Powellia* in New Zealand; see Martoni et al. [107]), and numerous instances of wide host switching have been suggested as a key process during the evolution of Psylloidea that enabled psyllids to colonize many different plant families [28]. More studies revealing the host plants of sister taxa will be needed to understand the occurrence rate of these apparently relatively rare wide host switching events in psyllids.”

Added reference: Martoni F, Bulman SR, Piper AM, Pitman A, Taylor GS, Armstrong KF (2023) Insect phylogeny structures the bacterial communities in the microbiome of psyllids (Hemiptera: Psylloidea)

in Aotearoa New Zealand. PLoS ONE 18(5): e0285587. doi: 10.1371/journal.pone.0285587

- Line 730: not sure if this is a requirement from the journal, but these references are not in order of publication.

**RESPONSE**: We have corrected the citation order.

=====
